# A comparison between the combined effect of calcium carbonate with sucroferric oxyhydroxide and other phosphate binders: an in vitro and in vivo experimental study

**DOI:** 10.1186/s12882-019-1655-9

**Published:** 2019-12-12

**Authors:** Atsushi Yaguchi, Kenji Akahane, Kumi Tsuchioka, Saori Yonekubo, Shota Yamamoto, Yasuaki Tamai, Satoshi Tatemichi, Hiroo Takeda

**Affiliations:** Pharmacology Research Group, Pharmacology and Pharmacokinetics Research Laboratory, R&D, Kissei Pharmaceutical Co., Ltd., 4365-1 Kashiwabara, Hotaka, Azumino, Nagano, 399-8304 Japan

**Keywords:** Hyperphosphatemia, Phosphate binders, Sucroferric oxyhydroxide, Calcium carbonate, Combined effects, Combination therapy

## Abstract

**Background:**

Approximately 30% of patients on dialysis received combination therapy for their phosphate binder prescription; however, few studies for combined effects of phosphate binders are reported. For the purpose of evaluating the efficacy of combination therapy, we compared the efficacy of sucroferric oxyhydroxide (PA21) combined with calcium carbonate with that of lanthanum carbonate hydrate, sevelamer hydrochloride, and ferric citrate hydrate combined with calcium carbonate.

**Methods:**

For in vitro studies, calcium carbonate and the other phosphate binders alone or in combination were stirred in phosphate solution at pH 2–8 for 2 h. After centrifuging the suspension, the phosphorus level in the supernatant was determined. For in vivo studies, rats were orally administered calcium carbonate and the other phosphate binders (except for sevelamer hydrochloride) alone or in combination, followed by oral administration of phosphate solution adjusted to pH 2 or 7. Serum samples were collected from the rats at predetermined timepoints and the serum phosphorus levels were determined and analyzed using a two-way analysis of variance.

**Results:**

In the in vitro study, the measured phosphate-binding capacity of combining sevelamer hydrochloride, PA21, and lanthanum carbonate hydrate with calcium carbonate was approximately equal to or greater than the theoretical values under most conditions. Furthermore, these combined effects were insensitive to pH in that order. The measured phosphate-binding capacity of ferric citrate hydrate combined with calcium carbonate was smaller than the theoretical values, and the combination did not exhibit efficacy under any of the tested conditions. In the in vivo study, the combined effect of PA21 and calcium carbonate at both pH values and that of lanthanum carbonate hydrate and calcium carbonate at pH 2 were additive. In contrast, the combined effect of lanthanum carbonate hydrate and calcium carbonate at pH 7 and that of ferric citrate hydrate and calcium carbonate at pH 2 were antagonistic.

**Conclusions:**

These results suggest that coadministration of PA21 and calcium carbonate showed good and relatively stable efficacy throughout the range of the gastrointestinal pH and that combining lanthanum carbonate hydrate and ferric citrate hydrate with calcium carbonate may not produce the expected efficacy under certain conditions.

## Background

In patients with end-stage kidney disease, renal function is reduced along with phosphorus excretion from the kidney, resulting in the accumulation of absorbed dietary phosphorus in the body and hyperphosphatemia [1–3]. Chronic kidney disease (CKD) is accompanied by systemic abnormalities such as laboratory test abnormalities, ectopic calcification, and bone disorder, which are collectively called CKD-mineral and bone disorder (CKD-MBD) and affect prognosis [4]. Serum phosphorus level has been reported to contribute the most to mortality among values of laboratory examination (serum phosphorus, calcium, and parathyroid hormone levels) recommended to be controlled within reference values for the treatment of CKD-MBD. Therefore, appropriate control of serum phosphorus level is required in the treatment of CKD-MBD [5, 6]. In clinical practice, restriction of dietary phosphate intake and phosphorus excretion with dialysis are performed to control serum phosphorus levels of patients on dialysis; however, these measures alone are not sufficient and phosphorus absorption from the gastrointestinal tract needs to be suppressed using oral phosphate binders [7].

Currently, oral phosphate binders such as calcium carbonate, sevelamer hydrochloride, lanthanum carbonate hydrate, ferric citrate hydrate, and sucroferric oxyhydroxide are used to treat hyperphosphatemia in Japan. Among these, calcium carbonate is universally used because of usage experience, efficacy, and much lower cost. However, the calcium carbonate dosage is restricted because calcium overload promotes coronary arterial and ectopic calcification and is associated with mortality [8–11]. As a countermeasure for these issues, calcium carbonate has been used in combination with other phosphate binders.

We previously reported that the effect of different pH values on the phosphate-binding capacity varied among phosphate binders [12]. In that study, calcium carbonate was one of the drugs with a phosphate-binding capacity that was susceptible to pH. Furthermore, since calcium carbonate is an alkaline drug with the associated physical property, the pH of the gastrointestinal tract may change after administration. This suggests that changes in the pH of the gastrointestinal tract due to calcium carbonate could affect the efficacy of other phosphate binders when they are coadministered with calcium carbonate. Additionally, other phosphate binders such as ferric citrate hydrate are acidic drugs, and, in contrast to the above, they might inhibit the efficacy of calcium carbonate.

Several studies have compared the phosphate-binding capacity of phosphate binders to date [12–14]. Furthermore, Cannata-Andía et al. have reported that the combined therapies with calcium-containing phosphate binders, sevelamer and lanthanum carbonate showed a beneficial association with survival in clinical practice [15]; however, there have been few detailed comparisons of phosphate-binding capacity in the combined therapies including new phosphate binders such as sucroferric oxyhydroxide. Therefore, for the purpose of evaluating the efficacy of combination therapy for phosphate binders, we examined the combined effect of sucroferric oxyhydroxide (PA21) and calcium carbonate in vitro and in vivo, and compared it with that of calcium carbonate in combination with other phosphate binders (lanthanum carbonate hydrate, sevelamer hydrochloride, and ferric citrate hydrate).

## Methods

### Drugs used

PA21 (Vifor Pharma, Glattbrugg, Switzerland), lanthanum carbonate hydrate (Alfa Aesar, Lancashire, UK), sevelamer hydrochloride (AK Scientific, Inc., Union City, CA, USA), calcium carbonate (FUJIFILM Wako Pure Chemical Corporation, Osaka, Japan), and ferric citrate hydrate (Nacalai Tesque, Inc., Kyoto, Japan) were used for the experiments. For the in vivo studies, each investigational drug was dissolved or suspended in 0.5% (w/v) methylcellulose (MC) solution (FUJIFILM Wako Pure Chemical Corporation). After phosphate (FUJIFILM Wako Pure Chemical Corporation), disodium hydrogen phosphate dodecahydrate (FUJIFILM Wako Pure Chemical Corporation), and sodium dihydrogen phosphate dihydrate (FUJIFILM Wako Pure Chemical Corporation) were diluted or dissolved in distilled water, the resulting phosphate solution was mixed and adjusted to pH 2, 3, 4, 5, 6, 7, or 8 [16], simulating the conditions in the digestive tract.

### Animals used

Male Sprague-Dawley (SD) rats (Charles River Laboratories Japan, Inc., Yokohama, Japan), 6–7 weeks of age and 140–250 g at arrival, were housed in groups of 3–5 per cage, and reared under constant temperature (20–26 °C), humidity (30–70%), and a 12 h light cycle (lights on at 8 AM, and off at 8 PM). All animals were provided with free access to water and a diet containing 1.08% (0.98–1.17%) phosphorus and 25.5% (24.6–26.3%) crude protein (CE-2 pellet, CLEA Japan, Inc., Tokyo, Japan). All animal experiments were performed in accordance with the guidelines approved by the Laboratory Animal Committee of Kissei Pharmaceutical Co., Ltd., which conform to the current Japanese laws.

### In vitro phosphate-binding capacity

#### Phosphate-binding capacity of various phosphate binders at pH 2–8

In 40 mL phosphate solution (20 mM = 1899 mg phosphate/L = 619 mg phosphorus/L), 200 mg PA21, lanthanum carbonate hydrate, sevelamer hydrochloride, calcium carbonate, or ferric citrate hydrate was suspended, and the resulting suspensions were stirred (37 °C, 2 h). The suspension was pelleted by centrifugation (37 °C, 2330×*g*, 5 min) after stirring, and the supernatant was collected. The phosphorus level in the supernatant was measured using spectrophotometry (SpectraMax, Molecular Devices Japan, K.K., Tokyo, Japan) by the molybdenum blue method (Phosphor C-Test Wako, FUJIFILM Wako Pure Chemical Corporation). The quantities of phosphate adsorbed onto 1 g of the ingredients in the investigational drug were determined as the phosphate-binding capacity of the drug. As shown in Table 1, the ingredients in PA21, lanthanum carbonate hydrate, calcium carbonate, and ferric citrate hydrate were regarded as the respective metals and that in sevelamer hydrochloride was regarded as sevelamer in this study.

**Table 1:** Content (%) of ingredients in each phosphate binder

Drug	PA21 (Sucroferric oxyhydroxide)	Lanthanum carbonate hydrate (LC)	Sevelamer hydrochloride (SH)	Calcium carbonate (CC)	Ferric citrate hydrate (FC)
Ingredient	Iron	Lanthanum	Sevelamer	Calcium	Iron
Content of ingredient	22%	52%	82%^a^	40%	18%

^a^Calculated using 831.5 as the molecular weight per repeating unit structure, which was calculated using the molecular formula of sevelamer hydrochloride [(C_3_H_7_N)_x_(C_9_H_18_ON_2_)_y_•nHCl]_z_ and the composition ratio of the repeating units of x = 9, y = 1, n = 4

#### Phosphate-binding capacity of calcium carbonate coadministered with each phosphate binder at pH 2–8

In 40 mL phosphate solution (20 mM), 100 mg calcium carbonate and 100 mg PA21, lanthanum carbonate hydrate, sevelamer hydrochloride, or ferric citrate hydrate were suspended. The resulting suspensions were stirred (37 °C, 2 h), pelleted by centrifugation (37 °C, 2330×*g*, 5 min) after stirring, and the supernatant was collected. The phosphorus level in the supernatant was measured using Phosphor C-Test Wako and SpectraMax in accordance with the method specified in the previous section, and the quantities of phosphate adsorbed onto 1 g of the total ingredients (the mixture of two ingredients, e.g., iron and calcium for PA21 and calcium carbonate used together) of the whole investigational drug for combination use was determined as the measured phosphate-binding capacity in combination use. Furthermore, the quantities of phosphate adsorbed in single drug, which were obtained in the previous section, and the composition (%) of each ingredient of the whole investigational drug in combination use (Table 2) were used to calculate the theoretical phosphate-binding capacity following combined use, according to the following formula.

$$ \mathrm{Theoretical}\ \mathrm{value}={\mathrm{Q}}_{\mathrm{C}\mathrm{C}}\times {\mathrm{C}}_{\mathrm{C}\mathrm{C}}+{\mathrm{Q}}_{\mathrm{PB}}\times {\mathrm{C}}_{\mathrm{PB}} $$

**Table 2:** Content (%) of ingredients and composition (%) of each ingredient in combination use

Drugs		CC + PA21	CC + LC	CC + SH	CC + FC
Ingredient		Calcium and iron	Calcium and lanthanum	Calcium and sevelamer	Calcium and iron
Content of ingredient^a^		31%	46%	61%	29%
Composition of each ingredient^b^	CC (C_CC_)	65%	43%	33%	69%
	PB (C_PB_)	35%	57%	67%	31%

^a^Calculated as the percentage of the weight of all the ingredients to that of the investigational drugs. ^b^Calculated as the percentage of the weight of CC or PB ingredient to that of all ingredients in investigational drugs. *CC* Calcium carbonate, *LC* Lanthanum carbonate hydrate, *SH* Sevelamer hydrochloride, *FC* Ferric citrate hydrate, *PB* Phosphate binder used in combination with calcium carbonate

Q_CC_: The quantity of phosphate adsorbed onto 1 g of the ingredient of calcium carbonate (calcium).

Q_PB_: The quantity of phosphate adsorbed onto 1 g of the ingredient of each phosphate binder (iron, lanthanum, or sevelamer,).

C_CC_: The composition (%) of the ingredient of calcium carbonate (calcium) to the ingredients of the whole investigational drug in combination use.

C_PB_: The composition (%) of the ingredient of each phosphate binder (iron, lanthanum, or sevelamer,) to the ingredients of the whole investigational drug in combination use.

### In vivo phosphate-binding capacity

#### Experimental design

The experiments were conducted at a total of six conditions for each combination of two experiment conditions: pH of phosphate solution administered (pH 2 or 7) and phosphate binder used in combination with calcium carbonate (PA21, lanthanum carbonate hydrate, or ferric citrate hydrate). The rats were randomly divided into the following five groups for each condition: (1) normal (MC solution + MC solution + distilled water), (2) control (MC solution + MC solution + phosphate solution), (3) calcium carbonate (calcium carbonate + MC solution + phosphate solution), (4) the other phosphate binder (PA21, lanthanum carbonate hydrate, or ferric citrate hydrate) (MC solution + the other phosphate binder + phosphate solution), (5) combination (calcium carbonate + the other phosphate binder + phosphate solution).

#### Effect of coadministration of each phosphate binder and calcium carbonate on serum phosphorus level after administration of phosphate solution to rats

Male SD rats were fasted for approximately 16 h and then restrained by hand without anesthesia to collect blood samples from the cervical vein (0 h measurements). Subsequently, calcium carbonate and other phosphate binders (PA21, lanthanum carbonate hydrate, or ferric citrate hydrate) alone or in combination were orally administered. Furthermore, a 0.5% MC solution was orally administered to the normal, control, and single drug-treated animals. Then, phosphate solution (200 mg/kg) adjusted to pH 2 or 7 was orally administered. The dose of each phosphate binder was set based on the dose [12] at which their suppression of increased serum phosphorus levels in the phosphate solution-loading rat model was confirmed. Blood samples were collected from the cervical vein 1, 2, 4, and 6 h after administration of each investigational drug. The collected blood samples were transferred to Capiject® collection tubes (Terumo Corporation, Tokyo, Japan), mixed by inverting, and kept for 30 min at approximately 23 °C. In a row on end, the level of phosphorus in serum obtained by centrifugation (4 °C, 2280×*g*, 10 min) was measured using the Phosphor C-Test Wako and SpectraMax in accordance with the method specified in the previous section. The rats that were used in the experiments were euthanized using the method of inhalation of carbon dioxide (CO_2_).

### Analysis

Each value is shown as the mean alone or ± standard error. The serum phosphorus level in rats is shown as the change (%) relative to the 0 h measurement, which was considered as 100%, and the area under the curve from 0 to 2 h (AUC_0–2 h_) was calculated as an evaluation item. The F-test was conducted to analyze differences between the normal and control groups in AUC_0–2 h_ for serum phosphorus level, and Student’s *t-*test (for equal variances) and Aspin-Welch’s *t*-test (for unequal variances) were conducted. A two-way analysis of variance (ANOVA) was used to analyze the effects of the interaction between each phosphate binder (PA21, lanthanum carbonate hydrate, and ferric citrate hydrate) and calcium carbonate on the serum phosphorus level. Significant differences were identified at *p* < 0.05 on a two-tailed basis.

## Results

### In vitro phosphate-binding capacity

#### Phosphate-binding capacity of each phosphate binder

**Fig. 1:**
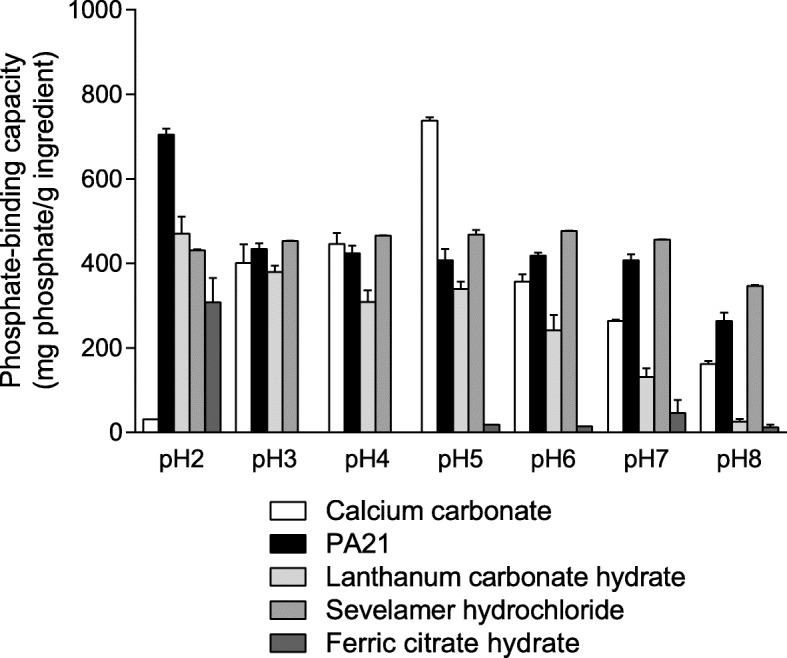
Phosphate-binding capacity of each phosphate binder. Each column in the figure shows the mean + standard error of three cases (two cases at pH 2 for calcium carbonate, and ND at pH 3 and 4, one case at pH 5 and 6, and two cases at pH 7 and 8 for ferric citrate hydrate, as no adsorption was observed in any or all cases). The phosphate-binding capacity of each investigational drug was calculated as the quantity of phosphate adsorbed onto 1 g of phosphate binder ingredient (metal or sevelamer). ND: Not detected

The quantities of phosphate adsorbed onto 1 g of the ingredient of the various phosphate binders at pH 2–8 are shown in Fig. 1. PA21 showed the highest phosphate-binding capacity at pH 2 of all tested pH levels, and retained relatively high binding capacity at pH 3–7. Lanthanum carbonate hydrate also showed the highest binding capacity at pH 2 of all the tested pH levels, and relatively high binding capacity at pH 5 and below. However, for lanthanum carbonate hydrate, the binding capacity decreased with increasing pH above 5, and phosphate adsorption was hardly observed at pH 8. Sevelamer hydrochloride showed almost the same phosphate-binding capacity at all tested pH levels. Ferric citrate hydrate showed phosphate-binding capacity at pH 2, but little or no phosphate adsorption was observed at pH 3–8. Calcium carbonate hardly showed any phosphate-binding at pH 2 and exhibited bell-shaped binding, which peaked at pH 5. The pH values 2 h after suspension are shown in Table 3. Calcium carbonate increased the pH of the solution by ≥1 at pH 2–5. Similarly, PA21, lanthanum carbonate hydrate, and sevelamer hydrochloride increased the pH of the solution at pH 3–5; however, sevelamer hydrochloride increased the pH level less than the other two drugs did at pH 3–5. Ferric citrate hydrate decreased the pH of the solution by ≥1 at pH 5–8.

**Table 3:** Last pH after suspension of each phosphate binder and calcium carbonate alone or in combination

Initial pH of solution	CC	PA21	LC	SH	FC	CC + PA21	CC + LC	CC + SH	CC + FC
2.0	5.3	2.3	2.3	2.2	2.1	5.6	6.3	5.7	4.4
3.0	6.5	6.2	6.4	4.9	3.0	6.8	6.9	6.3	5.0
4.0	6.8	6.5	6.5	5.5	3.1	6.9	7.1	6.3	5.0
5.0	7.3	6.6	6.7	5.7	3.1	7.0	7.2	6.3	5.0
6.0	6.8	6.7	6.7	6.0	3.2	7.0	7.0	6.5	5.1
7.0	7.3	7.4	7.2	7.6	3.7	7.5	7.3	7.4	5.9
8.0	8.7	8.9	8.1	8.5	4.2	8.9	8.7	8.6	6.7

*CC* Calcium carbonate, *LC* Lanthanum carbonate hydrate, *SH* Sevelamer hydrochloride, *FC* Ferric citrate hydrate

#### Phosphate-binding capacity of coadministered calcium carbonate and other phosphate binders

**Fig. 2:**
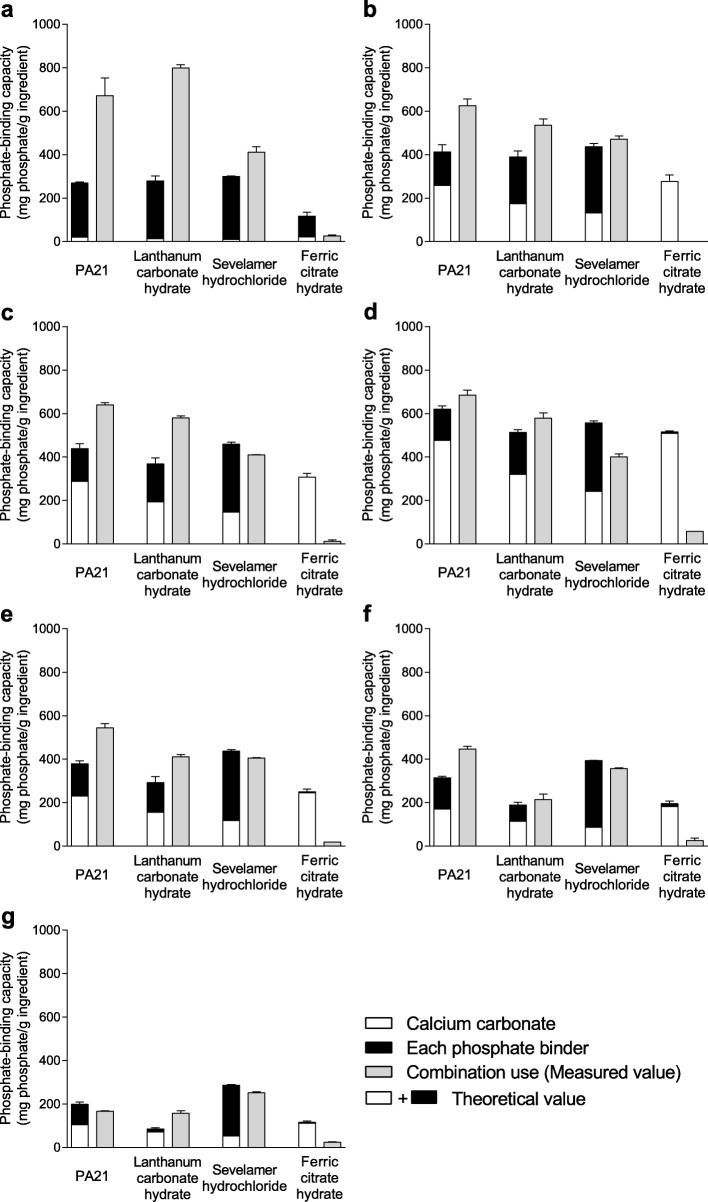
Comparison of theoretical and measured phosphate-binding capacity of each coadministration at pH 2–8. The phosphate-binding capacity at (**a**) pH 2, (**b**) pH 3, (**c)** pH 4, (**d**) pH 5, (**e**) pH 6, (**f**) pH 7, and (**g**) pH 8. Each column shows the mean + standard error of three cases (two cases for CC and CC + FC at pH 2, ND for FC and CC + FC at pH 3, ND for FC and two case for CC + FC at pH 4, one case for FC and CC + FC at pH 5, one case for FC and CC + FC at pH 6, two cases for FC at pH 7, and two cases for FC at pH 8, as no adsorption was observed in any or all cases). ND: Not detected

The comparison of the measured and theoretical quantities of phosphate adsorbed onto 1 g of the ingredient in combination with each phosphate binder and calcium carbonate at pH 2–8 are shown in Fig. 2. Furthermore, the measured phosphate adsorption quantities of each drug combination are shown in Table 4. The measured values following coadministration of PA21, lanthanum carbonate hydrate, and sevelamer hydrochloride with calcium carbonate were equal to or larger than the theoretical values at all tested pH levels, except for the combination with sevelamer hydrochloride at pH 5. Therefore, it was suggested that the effects of these combination uses were additive effects or above. In contrast, the measured values following coadministration of ferric citrate hydrate with calcium carbonate were smaller than the theoretical values at all tested pH levels, suggesting that the effect of this combination was antagonistic. In addition, the phosphate-binding capacities of the combination of PA21, lanthanum carbonate hydrate, and sevelamer hydrochloride with calcium carbonate were stable at pH 2–7, 2–6, and 2–8, respectively. The combination of ferric citrate hydrate with calcium carbonate hardly exhibited any binding capacity at all tested pH levels. Two hours after suspension, the pH value increased by ≥1 in the phosphate solution containing PA21 and lanthanum carbonate hydrate at pH 2, sevelamer hydrochloride at pH 2 and 3, and ferric citrate hydrate at all tested pH levels in combination with calcium carbonate, compared with that containing each phosphate binder alone (Table 3).

**Table 4:** Measured phosphate-binding capacity of coadministered calcium carbonate and each phosphate binder

Drugs	Phosphate-binding capacity (mg phosphate/g ingredient)			
	CC + PA21	CC + LC	CC + SH	CC + FC
pH 2	671 ± 81	799 ± 15	411 ± 25	26 ± 4
pH 3	625 ± 32	535 ± 29	471 ± 15	ND
pH 4	640 ± 11	579 ± 10	410 ± 1	11 ± 7
pH 5	684 ± 23	579 ± 24	401 ± 14	58
pH 6	544 ± 19	410 ± 11	405 ± 3	19
pH 7	446 ± 13	214 ± 25	357 ± 4	26 ± 12
pH 8	167 ± 3	157 ± 11	251 ± 6	24 ± 2

Each value shows the mean value ± standard error of three cases (two cases for CC + FC at pH 2, ND for CC + FC at pH 3, two cases for CC + FC at pH 4, one case for CC + FC at pH 5, and one case for CC + FC at pH 6 as no adsorption was observed in any or all cases). *CC* Calcium carbonate, *LC* Lanthanum carbonate hydrate, *SH* Sevelamer hydrochloride, *FC* Ferric citrate hydrate, *ND* Not detected

### In vivo phosphate-binding capacity

#### Effect of coadministration of various phosphate binders and calcium carbonate on serum phosphorus level after administration of phosphate solution to rats

**Fig. 3:**
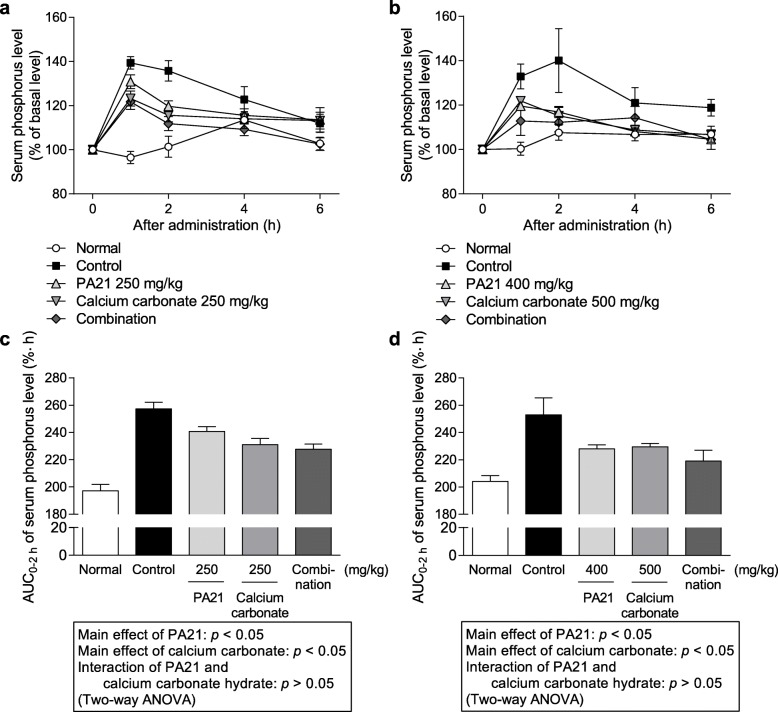
Serum phosphorus level following coadministration of PA21 and calcium carbonate. Temporal transition at (**a**) pH 2 and (**b**) pH 7. Area under the curve from 0 to 2 h (AUC_0–2 h_) of serum phosphorus level at (**c**) pH 2 and (**d**) pH 7. Each dot shows the mean value ± standard error, and each column shows the mean value + standard error of 7–12 cases at pH 2 (7 cases for normal, 11 cases for control, 12 cases for PA21, 11 cases for calcium carbonate, and 11 cases for combination) and 7–10 cases at pH 7 (10 cases for normal, 8 cases for control, 10 cases for PA21, 8 cases for calcium carbonate, and 7 cases for combination)

**Fig. 4:**
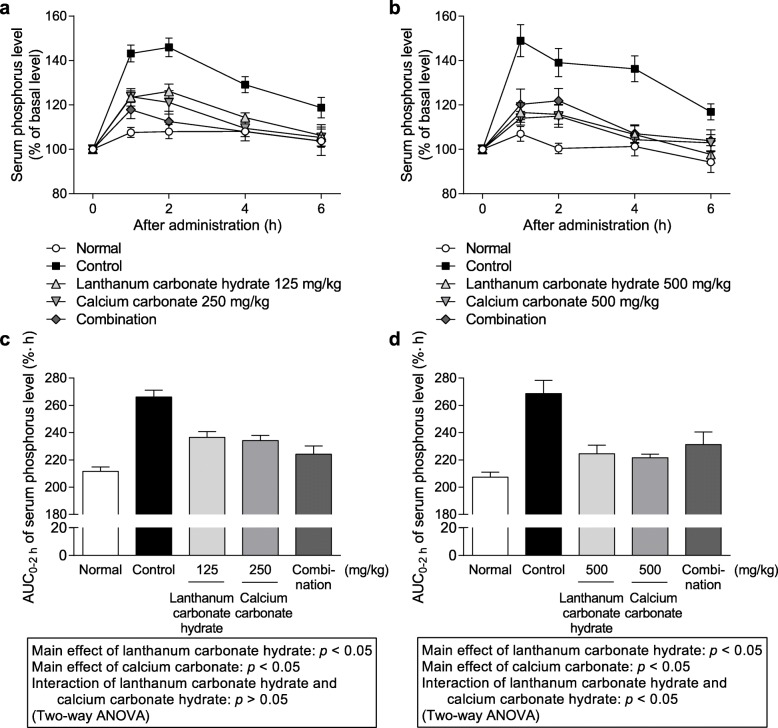
Serum phosphorus level following coadministration of lanthanum carbonate hydrate and calcium carbonate. Temporal transition at (**a**) pH 2 and (**b**) pH 7. Area under the curve from 0 to 2 h (AUC_0–2 h_) of serum phosphorus level at (**c**) pH 2 and (**d**) pH 7. Each dot shows the mean value ± standard error, and each column shows the mean value + standard error of 8–12 cases at pH 2 (12 cases for normal, 10 cases for control, 9 cases for lanthanum carbonate hydrate, 8 cases for calcium carbonate, and 8 cases for combination) and 10–12 cases at pH 7 (12 cases for normal, 11 cases for control, 12 cases for lanthanum carbonate hydrate, 10 cases for calcium carbonate, and 12 cases for combination)

**Fig. 5:**
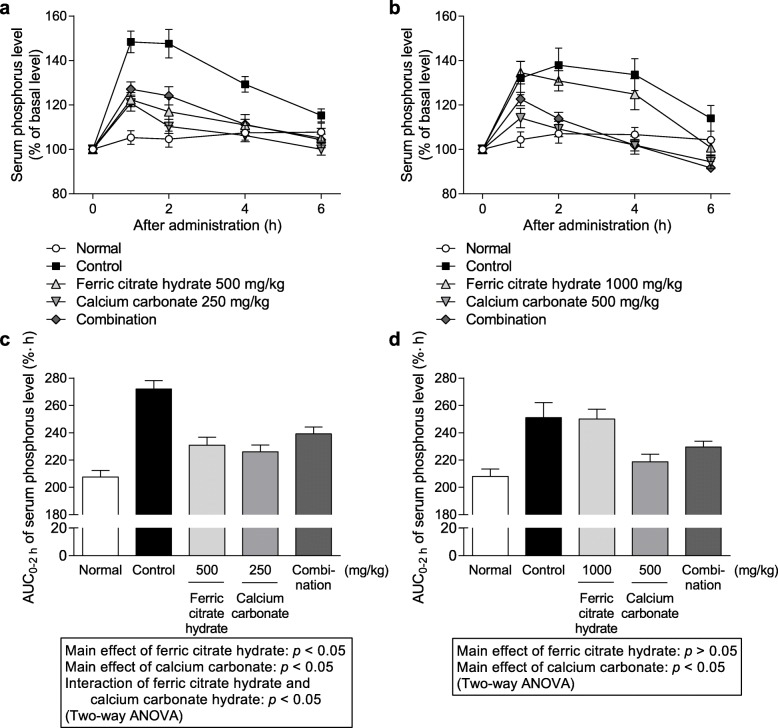
Serum phosphorus level following coadministration of ferric citrate hydrate and calcium carbonate. Temporal transition at (**a**) pH 2 and (**b**) pH 7. Area under the curve from 0 to 2 h (AUC_0–2 h_) of serum phosphorus level at (**c**) pH 2 and (**d**) pH 7. Each dot shows the mean value ± standard error, and each column shows the mean value + standard error of 9–12 cases at pH 2 (11 cases for normal, 12 cases for control, 11 cases for ferric citrate hydrate, 10 cases for calcium carbonate, and 9 cases for combination) and 9–12 cases at pH 7 (11 cases for normal, 10 cases for control, 12 cases for ferric citrate hydrate, 9 cases for calcium carbonate, and 11 cases for combination)

The effects of coadministration of PA21, lanthanum carbonate hydrate, and ferric citrate hydrate with calcium carbonate on temporal transition and AUC_0–2 h_ values of serum phosphorus level after the oral administration of phosphate solution (200 mg/kg) at pH 2 and 7 in rats are shown in Figs 3, 4 and 5, respectively. Coadministered PA21 (250 mg/kg) and calcium carbonate (250 mg/kg) at pH 2 exhibited significant main effects (*p* = 0.0131 and 0.0013, respectively), and there was no significant interaction (F_1, 43_ = 2.2284, *p* = 0.1428). Similarly, coadministration of PA21 (400 mg/kg) and calcium carbonate (500 mg/kg) at pH 7 showed significant main effects (*p* = 0.0167 and 0.0343, respectively), and there was no significant interaction (F_1, 29_ = 0.9751, *p* = 0.3316). Therefore, the combined effects of PA21 and calcium carbonate were additive at both pH values. Coadministration of lanthanum carbonate hydrate (125 mg/kg) and calcium carbonate (250 mg/kg) at pH 2 exhibited significant main effects (*p* = 0.0002 and 0.0001, respectively) and there was no significant interaction (F_1, 31_ = 4.0176, *p* = 0.0538). Furthermore, coadministration of lanthanum carbonate hydrate (500 mg/kg) and calcium carbonate (500 mg/kg) at pH 7 showed significant main effects (*p* = 0.0262 and 0.0223, respectively), and there was a significant interaction (F_1, 41_ = 12.0618, *p* = 0.0012). Therefore, the combined effects of lanthanum carbonate hydrate and calcium carbonate were additive at pH 2 and antagonistic at pH 7. Coadministration of ferric citrate hydrate (500 mg/kg) and calcium carbonate (250 mg/kg) at pH 2 showed significant main effects (*p* = 0.0061 and 0.0011, respectively), and there was a significant interaction (F_1, 38_ = 22.2665, *p* < 0.001). In contrast, calcium carbonate (500 mg/kg) showed a significant main effect (*p* = 0.0012) at pH 7 following coadministration with ferric citrate hydrate; however, no significant main effect of ferric citrate hydrate was observed at the high dose of 1000 mg/kg (*p* = 0.5341). Therefore, the combined effect of ferric citrate hydrate and calcium carbonate were antagonistic at pH 2 and were not evaluated at pH 7 because no significant main effect of ferric citrate hydrate was observed.

## Discussion

Hyperphosphatemia is one of the important risk factors which affecting mortality in dialysis patients [3, 17]. The control of serum phosphorus level is performed as treatment of hyperphosphatemia, through dietary phosphate restriction, dialysis, and oral administration of phosphate binders, and especially phosphate binders play a major role in the control of serum phosphorus levels [18, 19]. Various drugs are used as phosphate binders, and each drug has its own advantages, disadvantages, and adverse effects [19–21]. Adverse effects are usually associated with the administration of high-doses of phosphate binders; therefore, combination therapy is considered to contribute towards reducing these adverse effects and costs [7]. In clinical practice, it has been reported that a little under 30% of patients on dialysis received some kind of combination therapy for their phosphate binder prescription [22]. Especially, combination of calcium-based phosphate binders is estimated to be approximately 20% at minimum [23] and is the most common prescribing pattern in combination therapy. In Japan, calcium carbonate is the only approved calcium-based phosphate binder; therefore, it is considered that the majority of combination therapy prescriptions are combinations of calcium carbonate and other phosphate binders.

We examined the phosphate-binding capacity of combinations of calcium carbonate and other phosphate binders using in vitro studies, and the results indicated that some combination do not demonstrate the expected phosphate-binding capacity (theoretical phosphate-binding capacity; sum of efficacy of two binders). Among the combinations examined, the measured capacities of the combination of calcium carbonate with PA21 and lanthanum carbonate hydrate at pH 2 were approximately twice as high as the theoretical values. Regarding PA21, the rational reason was considered to be that, when calcium carbonate was coadministered with PA21, the phosphate-binding capacity of calcium carbonate was enhanced as the pH of the solution approached the pH at which calcium carbonate has the greatest efficacy. Moreover, the phosphate bound to PA21 at an acidic pH was retained even when the pH increased [24], which might also apply to lanthanum carbonate hydrate. Furthermore, enhancement of the phosphate-binding capacity differed between PA21 and sevelamer hydrochloride in combination with calcium carbonate, although the pH values were the same. This might be because the changes in pH immediately after suspension differed. The measured binding capacity of the combination of calcium carbonate with ferric citrate hydrate was lower than the theoretical value possibly because citrate ions chelate and decrease calcium ions, which can adsorb phosphate ions, and the pH at which the ferric citrate hydrate exerts phosphate-binding capacity was not maintained as coadministration with calcium carbonate increased the pH of the solution. Regarding the effect of pH on the phosphate-binding capacity of each combination except for that with ferric citrate hydrate, the phosphate-binding capacity of coadministered calcium carbonate and lanthanum carbonate hydrate at pH 7 and 8 and PA21 at pH 8 was less than half the values at pH 2–5. The phosphate-binding capacity of the coadministered calcium carbonate and sevelamer hydrochloride was the lowest at pH 8 of all tested pH conditions; however, even at pH 8, the capacity was approximately 50% of that at pH 2–5. This indicates that the phosphate-binding capacity following coadministration of calcium carbonate with sevelamer hydrochloride, PA21, and lanthanum carbonate hydrate was not affected by pH conditions in this order. This result was consistent with the insensitivity of the phosphate-binding capacity to pH with single use of phosphate binders [12].

The efficacy of coadministered calcium carbonate and each phosphate binder at pH 2 and 7 was investigated using the phosphate solution loading model previously reported as an in vivo evaluation system [12]. Although pH 2 is lower than the pH conditions in the stomach [16], since the phosphate-binding capacities of ferric citrate hydrate was severely diminished at pH ≥ 3 in the in vitro study, this pH was set so that ferric citrate hydrate could exert its efficacy. We used pH 7 to simulate the pH conditions of the upper and middle intestine, which is the main site of Na-independent inorganic phosphate absorption [25, 26]. Referring to our previous study [12], the doses of phosphate binders were set as the doses at which the main effects of each factor were observed using a two-way ANOVA to analyze the AUC_0–2 h_ of serum phosphorus level. However, at pH 7, ferric citrate hydrate was examined at the dosage at which the main effects were not observed, because this drug did not suppress the increased serum phosphorus level even at a high dose of 1000 mg/kg and a higher dose was considered unreasonable. The result of the two-way ANOVA indicated that the combined effect of calcium carbonate with PA21 at both pH values and with lanthanum carbonate hydrate at pH 2 were additive, and the combined effect of calcium carbonate with lanthanum carbonate hydrate at pH 7 and with ferric citrate hydrate at pH 2 were antagonistic. It is not clear why coadministered lanthanum carbonate hydrate and calcium carbonate did not show efficacy at pH 7. However, coadministration of lanthanum carbonate hydrate and calcium carbonate might create carbonate species-rich conditions, resulting in the reduced solubility of calcium carbonate and inhibition of the phosphate-binding capacity of calcium carbonate. This notion is based on reports that the solubility of calcium carbonate declines under carbonate species-rich conditions at pH 7.5 [27]. Therefore, examination of the coadministration of calcium carbonate and each phosphate binder showed that the result of the in vitro study mostly reflected that of the in vivo study, except with coadministration of calcium carbonate and lanthanum carbonate hydrate at pH 7. Furthermore, the combined effect of PA21 and calcium carbonate was retained even at changing pH condition in vivo [16, 28, 29].

It is not clear why the efficacy of coadministered lanthanum carbonate hydrate and calcium carbonate at pH 7 in the in vivo study differ from that in the in vitro study; however, there is a possibility that the change in pH affected the phosphate-binding capacity, resulting in unforeseen consequences, since the pH changed in the transition from the stomach to intestine in the in vivo study. Furthermore, phosphate administered in solution is absorbed relatively quickly into the body [30], whereas calcium carbonate and lanthanum carbonate hydrate are considered to have a slower rate of phosphate adsorption than PA21 [13]. Therefore, there is also the possibility that phosphate was absorbed into the body before the binders adsorbed phosphate and, consequently, lanthanum carbonate hydrate and calcium carbonate were not efficacious. The above observations suggest that the efficacy of phosphate binders might be limited under the conditions used in this study where phosphate loading by solution may cause more rapid changes in pH and quicker absorption of phosphate into the body than those occurring clinically. However, the combined effect of PA21 and calcium carbonate was stable even in this study and, thus, a stable combined effect can be expected to also be exerted in clinical settings, wherein the conditions would be more suitable for phosphate binders.

Gastric secretory inhibitors such as proton pump inhibitors and histamine H2-receptor antagonists are known to affect the gastrointestinal pH and are used to treat peptic ulcer and gastroesophageal reflux disease and eradicate *Helicobacter pylori*. It has been reported that the key to successful treatment is to maintain the intragastric pH at ≥3, ≥ 4, and at the neutral conditions, during each treatment [31–33], and these drugs are considered to increase the intragastric pH to these levels [34–36]. These drugs have been reported to be prescribed to approximately 38% of hemodialysis patients [37]; therefore, considerable number of patients are presumed to use gastric secretory inhibitors with phosphate binders. Moreover, the serum phosphorus lowering effect of calcium carbonate has been reported to be attenuated by administering these drugs [38, 39]. The same phenomenon has not yet been reported with other phosphate binders and there is a possibility that gastric secretory inhibitors attenuate their efficacy. However, in this study, PA21 exhibited a stable phosphate-binding capacity that was unsusceptible to pH both as a single-drug and in combination with calcium carbonate. This indicates that PA21 may control serum phosphorus levels adequately even when coadministered with prescribed gastric secretory inhibitors, which is an interesting consideration.

## Conclusion

In this experimental study, sevelamer hydrochloride, PA21, and lanthanum carbonate hydrate exhibited efficacy that was equal to or greater than the theoretical efficacy when coadministered with calcium carbonate. Furthermore, the efficacy of these drugs was shown to be insensitive to pH and adequately retained as both single-drug treatments or coadministered with calcium carbonate, in this order. In contrast, the combination of ferric citrate hydrate and calcium carbonate hardly showed any efficacy regardless of pH conditions. These results suggest that PA21 exhibited good and stable efficacy in combination with calcium carbonate at the gastrointestinal pH range and can be expected to show stable efficacy with changes in pH conditions of the gastrointestinal tract induced by other drugs. However, it is possible that lanthanum carbonate hydrate and ferric citrate hydrate in combination with calcium carbonate cannot exhibit the expected efficacy under certain pH conditions (Table 5).

**Table 5:** Combined effect expected in clinical practice

Drugs	pH 2 (Normal condition)	pH 7 (Condition simulating increased gastrointestinal pH)
CC + PA21	↑	↑
CC + LC	↑	↓
CC + FC	↓	–

*CC* Calcium carbonate, *LC* Lanthanum carbonate hydrate, *FC* Ferric citrate hydrate. ↑: Additive effect, ↓: Antagonistic effect, −: The combined effect was not evaluated because the main effect of ferric citrate hydrate was not observed

## Data Availability

All data generated or analysed during this study are included in this published article.

## Abbreviations

ANOVA: Analysis of variance
AUC_0–2 h_: Area under the curve from 0 to 2 h
CKD: Chronic kidney disease
CKD-MBD: Chronic kidney disease-mineral and bone disorder
MC: Methylcellulose
SD: Sprague-Dawley
